# Evidence for Dietary Fibre Modification in the Recovery and Prevention of Reoccurrence of Acute, Uncomplicated Diverticulitis: A Systematic Literature Review

**DOI:** 10.3390/nu10020137

**Published:** 2018-01-27

**Authors:** Camilla Dahl, Megan Crichton, Julie Jenkins, Romina Nucera, Sophie Mahoney, Wolfgang Marx, Skye Marshall

**Affiliations:** 1 Faculty of Health Sciences & Medicine, Bond University, Robina, QLD 4226, Australia; camilla.dahl@student.bond.edu.au (C.D.); mcrichto@bond.edu.au (M.C.); Sophie.mahoney@student.bond.edu.au (S.M.); wmarx@bond.edu.au (W.M.); 2 Department of Nutrition and Dietetics, Robina Hospital, Robina, QLD 4226, Australia; juliejenkins11@gmail.com (J.J.); Romina.Nucera@health.qld.gov.au (R.N.); 3 School of Allied Health, La Trobe University, Melbourne, VIC 3086, Australia

**Keywords:** dietary fibre, diverticulitis, diverticulosis, diverticular disease, systematic review, bowel rest, dietary restriction, probiotic

## Abstract

In practice, nutrition recommendations vary widely for inpatient and discharge management of acute, uncomplicated diverticulitis. This systematic review aims to review the evidence and develop recommendations for dietary fibre modifications, either alone or alongside probiotics or antibiotics, versus any comparator in adults in any setting with or recently recovered from acute, uncomplicated diverticulitis. Intervention and observational studies in any language were located using four databases until March 2017. The Cochrane Risk of Bias tool and GRADE were used to evaluate the overall quality of the evidence and to develop recommendations. Eight studies were included. There was “very low” quality evidence for comparing a liberalised and restricted fibre diet for inpatient management to improve hospital length of stay, recovery, gastrointestinal symptoms and reoccurrence. There was “very low” quality of evidence for using a high dietary fibre diet as opposed to a standard or low dietary fibre diet following resolution of an acute episode, to improve reoccurrence and gastrointestinal symptoms. The results of this systematic review and GRADE assessment conditionally recommend the use of liberalised diets as opposed to dietary restrictions for adults with acute, uncomplicated diverticulitis. It also strongly recommends a high dietary fibre diet aligning with dietary guidelines, with or without dietary fibre supplementation, after the acute episode has resolved.

## 1. Introduction

Diverticulitis is characterised by the acute inflammation and/or infection of diverticula in the colonic wall, often managed by hospitalisation for administration of intravenous antibiotics, dietary fibre modifications and monitoring [1,2]. The incidence, prevalence, and number of hospital admissions are rapidly increasing due to a rising prevalence of risk factors such as ageing, increasing central adiposity, sedentary lifestyles, and low diet quality [3,4,5]. Outpatient visits to physicians for diverticular disease are over 2.5 million annually in the United States of America [6], and the 300,000 annual hospital admissions for acute diverticulitis alone has a financial burden of USD$2.5 billion (£1.9 billion) [7]. Therefore, it is the timely treatment, prevention of reoccurrence of acute episodes, and decreased gastrointestinal symptoms which are important for improving patient and healthcare outcomes worldwide [8,9].

Upon diagnosis of acute, uncomplicated diverticulitis (i.e., no perforation, non-localised abscess, drains placed, or surgery required), short-term low dietary fibre intake or food deprivation for bowel rest is often used in clinical practice as it is thought that a less active bowel reduces colonic irritation and re-inflammation [10,11]. These treatment options are usually administered in the inpatient setting, but may also be provided as outpatient care. The efficacy of these treatment approaches in acute, uncomplicated diverticulitis have not yet been reviewed, and dietary fibre restrictions (herein referred to as a “restricted diet”) may be associated with longer hospital stay, increased patient burden, increased risk of malnutrition especially in older adults, and increased health care costs [12,13,14]. In addition to delaying recovery, there is evidence suggesting bowel rest in acute diseases of the colon is ineffective in reducing inflammation, risk of infection and other complications [15,16].

Following resolution of acute symptoms, patients are frequently recommended to follow a low dietary fibre diet and then transition to a standard or high dietary fibre diet (defined as meeting or exceeding national nutrient recommendations for age and gender) [17,18]. A high dietary fibre diet is hypothesised to prevent diverticulitis reoccurrence due to reducing the contact time between gut contents and diverticula, and the associated irritation [19,20,21]. There has also been increasing use of probiotic supplementation in practice, which is thought to decrease risk of infection and inflammation of the diverticula [22]. However, this practice has not yet been evaluated by a systematic literature review. A 2012 systematic review found no intervention studies which modified dietary fibre intake to prevent acute diverticulitis reoccurrence [23]. A second, more recent systematic review examined the efficacy of dietary fibre modifications for management of symptomatic uncomplicated diverticular disease (SUDD); however, did not address management of acute diverticulitis, a complication of SUDD [24]. The lack of information about what dietary recommendations should be given to patients with acute diverticulitis is reflected by inconsistent guidelines [1,2], and is likely to have important clinical implications, including increased patient burden and health service use [10].

Therefore, this study aims to systematically review observational and intervention evidence and develop preliminary recommendations for dietary fibre modifications, either alone or alongside probiotics and/or antibiotics, versus any comparator on recovery, reoccurrence, gastrointestinal symptoms and health care use for adults during or following an episode of acute, uncomplicated diverticulitis in any health care setting. Based on an exploratory literature review, hypotheses are that a liberalised diet is equal to a restricted diet for the outcomes of recovery, gastrointestinal symptoms, and reoccurrence, and superior in regards to health care use and costs. It is also hypothesised that after an acute episode has resolved, high dietary fibre intake (i.e., meeting nutrient reference values and dietary guidelines) and/or probiotic supplementation is superior to a standard (i.e. returning to the individual’s previous diet) or low fibre diet in regards to reoccurrence, gastrointestinal symptoms and health care use.

## 2. Materials and Methods

This systematic review was reported according to the Preferred Reporting Items for Systematic Reviews and Meta-Analyses (PRISMA) statement [25]. The study protocol was registered with the International Prospective Register of Systematic Reviews (PROSPERO Number: CRD42016048741).

### 2.1. Study Search and Selection

The electronic databases Medline (PubMed), Embase, Web of Science and CINAHL were searched for published literature in any language from database inception until the 31 March 2017, using a combination of keywords and controlled vocabulary adapted for each database. The full search strategy is shown in Online Supplementary Material 1. Briefly, the approach used for the search strategy was based on the following: (dietary fibre OR fibre OR carbohydrates OR starch OR diet* OR nutrition* OR residue OR roughage OR prebiotic* OR fasting OR food deprivation OR bowel rest OR nil by mouth OR conservative) AND (Diverticu*). 

A snowballing strategy was also used whereby reference lists of included studies, reviews and/or guideline documents were searched to identify additional studies not found in the search strategy; and a brief targeted search of Google Scholar was conducted.

Studies were included with adults (≥18 years) diagnosed with acute, uncomplicated diverticulitis, characterised by the absence of large or pelvic abscess, fistula, stricture, peritonitis, sepsis and surgery. Patients were included if interventions were implemented during the acute episode, or in the post-acute period (directly after acute inflammation and pain has subsided and the case is considered treated) to manage ongoing symptoms and prevent reoccurrence. Studies were included only where participants received dietary or supplemental fibre modifications (restriction or increase), including the implementation of bowel rest or liquid diets, with or without probiotic supplementation or antibiotic administration, in the any health care setting (inpatient or outpatient). Study populations that had diverticular disease or diverticulosis, without an episode of acute, uncomplicated diverticulitis, were excluded from this review and were reported elsewhere [26]. As there are very few randomised controlled trials examining the research questions, eligible study designs included prospective and retrospective intervention studies of any design and observational longitudinal studies. Ineligible study designs were cross-sectional studies, reviews, abstracts, study protocols, and conference papers, or those that did not report on any outcome of interest.

Eligible studies were then selected in a two-stage process. At Stage 1, the titles and abstracts of identified studies were screened for eligibility by two independent researchers (CD and MC). At Stage 2, the full text of potentially eligible articles were retrieved and independently reviewed for eligibility by two researchers (CD and MC), with the final selection discussed and agreed upon by consensus. 

### 2.2. Outcome Measures

To reflect the management of an acute episode of uncomplicated diverticulitis, hospital length of stay (LOS) was considered the primary outcome variable of interest; and secondary outcomes were diverticulitis reoccurrence, direct health service costs, recovery (indicated by incidence of treatment failures; defined as no clinical improvement with therapy or development of complications), quality of life, and gastrointestinal symptoms. Outcome measures were considered 24-h after presentation to five-years post-diagnosis.

To reflect management of acute, uncomplicated diverticulitis after the acute episode has resolved (absence of clinical signs and symptoms within any timeframe from initial acute episode), the primary outcome of interest was reoccurrence of acute diverticulitis (uncomplicated or complicated) within five years. Secondary outcomes were health care costs, quality of life, gastrointestinal symptoms, and outpatient medical visits regarding gastrointestinal concerns. Primary and secondary outcomes were considered from the resolution of the initial acute episode to five-years post episode.

Data relating to the sample population, intervention, comparator, and reported findings were extracted and reported qualitatively. The data extraction was conducted independently by two researchers (CD and MC), where all included studies were checked for accuracy by a senior researcher (S Marshall). Where continuous data was not reported as means and standard deviations, Review Manager was used to compute these (Review Manager (RevMan) [computer program]. Version 5.3. Copenhagen, Denmark: The Nordic Cochrane Centre, The Cochrane Collaboration, 2014).

### 2.3. Study Strength and Quality of the Evidence

Internal study quality of all included studies was assessed using the Cochrane risk of bias tool independently by two researchers (CD and MC); and reviewed for accuracy by a senior researcher (S Marshall) [27]. The certainty in the body of evidence for each outcome of interest and the development of recommendations for populations were developed using the Grading of Recommendations, Assessment, Development and Evaluation (GRADE) tool [28], following steps and interpretation as specified in the GRADE Handbook [29] and implemented using the software GRADEpro GDT (GRADEpro Guideline Development Tool, McMaster University, 2015, developed by Evidence Prime, Inc. Hamilton, Canada). Determination of the GRADE level of evidence was determined independently by two authors (CD and S Marshall), with disagreements managed by consensus. The factors that determine the direction and strength of recommendations for the clinical question considered a balance between desirable and undesirable outcomes, confidence in the magnitude of estimates of effect, confidence in values and preferences of stakeholders and the resources/feasibility of the recommendations [29]. The recommendations and justifications were developed in panel discussions with all authors of this review. 

### 2.4. Meta-Analysis

Meta-analysis was unable to be performed due to high heterogeneity among included studies. 

## 3. Results

**Figure 1:**
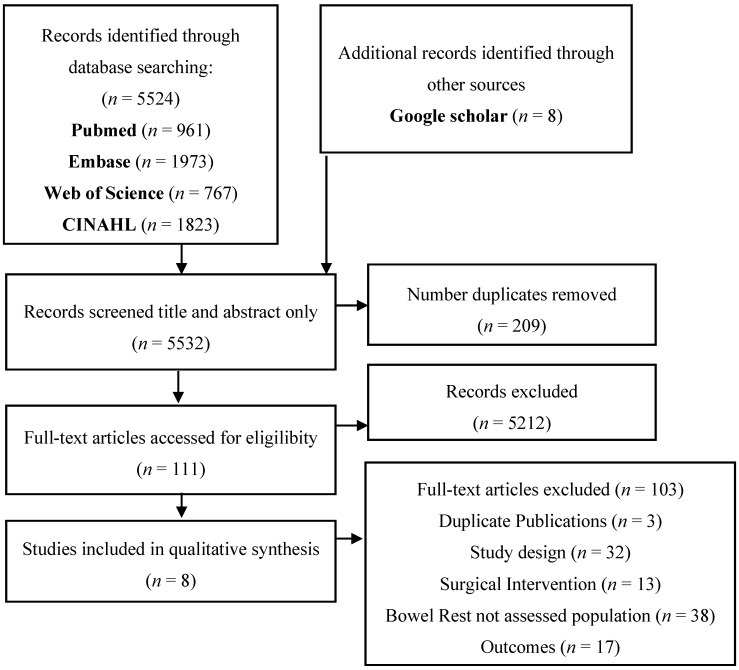
PRISMA flowchart of the search results and the included studies.

The electronic search identified 5524 records, where snowball searching identified a further eight studies (Figure 1). There were 111 papers retrieved for full text review, with 8 included in the qualitative analysis (Table 1andTable 2).

**Figure 2:**
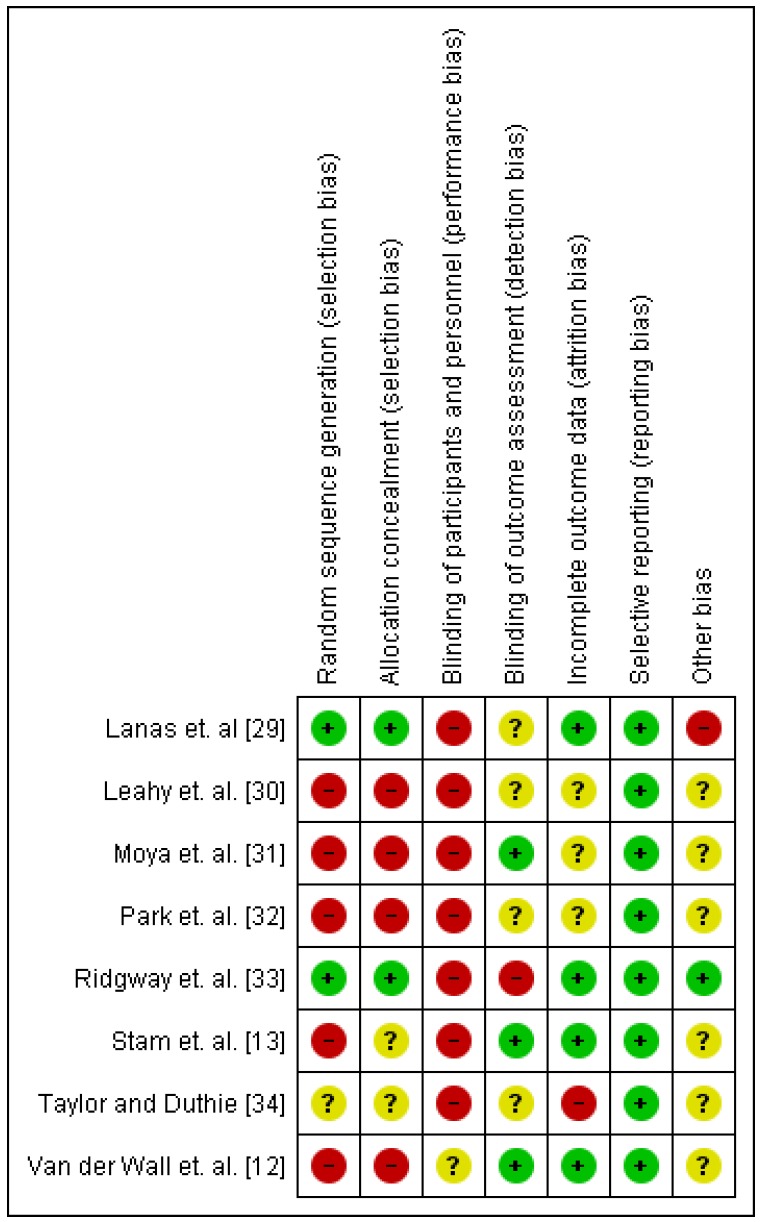
Cochrane risk of bias summary: review authors’ judgements about each risk of bias item for each included study.

All but two studies included had unclear or high risk of bias for four or more of the seven risk of bias domains (Figure 2). The most common high risk of bias was due to inadequate randomisation and blinding of participants (Online Supplementary Material 2). 

### 3.1. Liberalised versus Restricted Diets, with or without Antibiotics, for the Management of Acute, Uncomplicated Diverticulitis in the Inpatient and Outpatient Setting

Five studies (with eight comparator groups) were identified that reported the effects of dietary fibre modifications in the treatment of an acute episode of uncomplicated diverticulitis, either alone or in combination with other interventions [12,30,31] (Table 1). Three studies compared dietary fibre modifications via a randomised controlled trial [RCT] and two via observational studies. All groups were administered either oral or intravenous antibiotics in an inpatient setting, excepting the liberalised diet groups by Park et al. [30] and Moya et al. [32] in which participants were outpatients. Both groups in the study by Moya et al. [32] were given a restricted diet, and the studies by Park et al. [30] and Moya et al. [32] varied the types of antibiotic given to patients, where intravenous antibiotics were given to inpatients and oral antibiotics given to outpatients. Stam et al. [13] also examined participants in the outpatient setting; however, only one liberalised diet group was observed. No studies that examined the treatment of acute, uncomplicated diverticulitis reported use of any intervention to prevent reoccurrence after the treatment of the acute episode.

All studies reported hospital LOS; however, they could not be pooled due to poor reporting of data, as well as a large skew of the LOS in most studies. However, as expected, LOS was reported to be lower for liberalised diets compared to restricted diets in most studies. Although the RCT by Ridgway et al. [34] found no significant decrease in LOS with aliberalised diet, this outcome may have been underpowered given the wide standard deviation of the restricted diet group. The quality of the evidence (GRADE) for hospital LOS was “very low” (Online Supplementary Material 3).

Both Park et al. [30] and Ridgway et al. [31] evaluated the impact of liberalised versus restricted diets for the treatment of acute, uncomplicated diverticulitis on reoccurrence from 1 to 21 months post discharge. It was concluded that there was no significant difference between liberalised and restricted diets for reoccurrence, however, lack of consistent study design prevented data pooling and meta-analysis.

Five comparisons evaluated the risk of treatment failures, from three studies: Park et al. [30], Ridgway et al. [31], and three comparisons from van der Wall et al. [12], all of which reported low incidence of treatment failures (<4 per group). There was large heterogeneity between studies, however, based on the available evidence, there appears to be no difference in treatment failures in the liberalised compared to the restricted diet groups.

The quality of the body of evidence comparing liberalised and restricted diets in regards to both reoccurrence and recovery were assessed as “very low” (Online Supplementary Material 3). Only one study reported on gastrointestinal symptoms comparing liberalised and restricted diets, finding no difference between groups, with the quality of evidence being “very low” (Online Supplementary Material 3).

### 3.2. Dietary Fibre Modifications with or without Probiotic Supplementation, for the Management of Uncomplicated Diverticulitis after the Acute Episode Has Resolved

Three studies were identified that modified dietary fibre intake following an episode of acute, uncomplicated diverticulitis (Table 2), including the study by Taylor and Duthie [35] that used three intervention arms with no control group. No studies were identified that included probiotic supplementation alone or in addition to dietary fibre modifications. Although all studies reported on gastrointestinal symptoms and two reported on reoccurrence; no data could be pooled due to the absence of low dietary fibre control groups in two of the three studies. The body of evidence regarding a high dietary fibre diet and/or dietary fibre supplementation for both the prevention of reoccurrence and improving gastrointestinal symptoms is “very low” (Online Supplementary Material 3).

## 4. Discussion

This systematic review found a lack of high quality interventional research examining the dietary management of adults with acute, uncomplicated diverticulitis. However, the outcomes that could be evaluated by observational and/or lower quality intervention research tended to agree with the hypotheses that a liberalised and restricted diet are equal in terms of recovery (both having very low risk and incidence of treatment failures), reoccurrence, and gastrointestinal symptoms, with liberalised diets tending to have lower health care use. Therefore, this review found that available evidence suggests liberalised diets for inpatient treatment are safe in uncomplicated cases. The “very low” quality of the evidence comparing liberalised diets and restricted diets demonstrates that there is no existing research showing any clinical benefit to implementing a diet restriction; and identified no studies supporting the hypothesis that bowel rest is required for resolution of an acute episode in uncomplicated cases. The interpretation of findings was limited by high clinical heterogeneity, and results appear to be confounded by other intervention factors, such as differing antibiotics administration. Although one study was identified that used probiotics, it was not included as modifications to dietary fibre intake were not described, and the probiotics were prescribed to both intervention groups at the same dosage with only medications varied for comparison [22]. 

Despite a high risk of bias across most studies, and few studies examining the research questions, the GRADE approach as outlined by Guyatt et al. [37] was used to review the quality of the body of evidence and produce recommendations, which are discussed and justified briefly here. Research evidence, discussion and detailed justifications for judgements supporting these recommendations can be found in Online Supplementary Material 3.

### 4.1. Summary of Judgements and Recommendations for Liberalised versus Restricted Diets for the Inpatient Dietary Management of Acute, Uncomplicated Diverticulitis

#### 4.1.1. Recommendation for the Population

Adult patients admitted to hospital with acute, uncomplicated diverticulitis (i.e., no perforation, non-localised abscess, drains placed or surgery required) should be placed on a liberalised diet (i.e., allowing consumption of solid food) and not placed on a restricted diet (i.e., bowel rest/nil by mouth, clear and/or liquid diets). 

#### 4.1.2. Strength of the Recommendation

Conditional recommendation for the intervention (liberalised diets) based on a very low-quality body of evidence.

#### 4.1.3. Overall Justification

This review identified no evidence of a difference between liberalised and restricted diets in terms of clinical outcomes including recovery (treatment failures), reoccurrence or patient symptoms; however, liberalised diets may decrease length of hospital stay and prevent restriction of essential nutrient intake (e.g., dietary fibre, vitamins, minerals, phytonutrients, energy and protein found in solid foods) in patients. It can be generally accepted that the majority of patients would prefer autonomy and/or not to have food restrictions (such as nil by mouth or liquid only diets) prescribed unless there is evidence of a medical contraindication. It can also be generally accepted that health care providers would rather not place further nutrition restrictions on patients, which may require increased dietary management by physicians, dietitians and/or nursing staff. Placing patients on a liberalised diet is highly feasible. However, due to the poor quality and small amount of literature examining this research question, this recommendation was conditional based on a low quality of evidence. It should be highlighted that existing evidence suggests using a liberalised diet has a low risk of harm and likely benefits to patients and the health care system. Despite a recommendation in favour of liberalised diets, health care providers should consider the individual risk profiles of patients to identify other potential contraindications for oral intake, such as co-morbidities or risk for the development of complications (i.e., presence of bleeding, abscess or perforation).

#### 4.1.4. Detailed Justification:

*Values*: It is generally accepted that patients and health care providers do not wish to impose unnecessary dietary restrictions, which may cause patient and health care provider burden, increased discomfort, decreased appetite, decreased nutrient intake, and increased risk of malnutrition for older adults. It is generally accepted that decreased length of hospital stay is desirable for patients, health care providers and health services.

*Resources required*: There are no resources required to implement the intervention; and there is likely to be a saving of resources (staff time) by not implementing a restricted diet which can involve multiple types of diet restrictions and which requires ongoing monitoring and evaluations.

*Cost effectiveness*: There is no monetary cost to implement the intervention, but likely increased costs to implement the comparison (restricted diet) through increased health care provider management of dietary restrictions, and/or increased risk of complications associated with nutrient/food deprivation (e.g., malnutrition).

*Acceptability*: A liberalised diet is likely to be acceptable to patients and health care providers. Liberalised diets are not forcing the patient to eat if they do not wish to; it is simply offering autonomy to consume food, allows more food options and minimises struggles over dietary compliance. It is likely to require less health care resources and impose less burden on patients.

*Subgroup considerations*: Restriction of dietary intake in older adults (≥65 years), patients with increased nutrient requirements, and those with food insecurity (e.g., financial insecurity or drug/alcohol-dependence) should be minimised as much as possible due to their significantly higher risk of malnutrition.

*Implementation considerations*: There are minimal implementation considerations. Implementation of the intervention is simply not imposing any dietary restrictions.

*Monitoring and evaluation*: Patients should be monitored closely, as per current recommendations, for signs of worsening condition and/or progression to complicated diverticulitis.

### 4.2. Summary of Judgements and Recommendations for a High Dietary Fibre Diet versus Low Dietary Fibre or Standard Diets for the Dietary Management of Uncomplicated Diverticulitis after the Acute Episode Has Resolved

#### 4.2.1. Recommendation for the Population

Health care providers should recommend a long-term high dietary fibre intake (meeting the nationally recommended intake for gender and age) after the acute episode of uncomplicated diverticulitis has resolved.

#### 4.2.2. Strength of the Recommendation

Strong recommendation for the intervention based on very low-quality body of evidence.

#### 4.2.3. Overall Justification

This review found low confidence in the evidence that high dietary fibre intake will result directly in improved risk for diverticulitis reoccurrence and/or gastrointestinal symptoms; but also found no evidence supporting the use of a low dietary fibre diet. A high dietary fibre diet is recommended as the standard diet for all adults by dietary guidelines [38,39], and therefore this recommendation stands even though there is no strong confidence in added benefit for diverticulitis-related outcomes. This recommendation is considered strong, based on very low-quality-evidence, as potential benefits clearly outweigh risk, and support implementation of dietary guidelines [37]. Furthermore, a strong recommendation based on very low quality of evidence aligns with recommendations made by the American Society of Colon and Rectal Surgeons (ASCRS) for the medical management of acute diverticulitis [17]. It should be recognised that achieving sustained dietary change is difficult in western societies as it is not supported by the food environment [40], and therefore this diet should be recommended along with long-term support to achieve dietary change [41]. The evidence shows some improvement in clinical outcomes with the use of dietary fibre supplements with or without food-based dietary fibre increases; however, there is insufficient evidence to make specific supplementation recommendations [38,42]. Some dietary guidelines do recommend nutrient supplementation in general if the nutrient target cannot be met through diet alone; therefore, this option should be considered on an individual basis [38]. Additionally, some patients have an intolerance to some forms of dietary fibre and/or other comorbidities which require a limitation or modification of dietary fibre intake [43]. These patients should receive individualised advice with follow-up to help achieve the best outcomes and management of potential ongoing symptoms.

#### 4.2.4. Detailed Justification

*Subgroup considerations*: People with low-socioeconomic backgrounds are likely to require further support to achieve a high dietary fibre diet as this sub-group may have a poorer quality of diet at baseline, and have less resources to access dietary support services [44]. A high dietary fibre intake or specific high fibre foods may also be contraindicated in patients with additional comorbidities, particularly other diseases of the gastrointestinal tract or kidneys; and therefore, recommendations for these subgroups should be individualised. 

*Implementation considerations*: Although the recommendation of achieving a high dietary fibre diet may easily be made, patients may not be aware of what constitutes a high fibre diet and how it can be achieved [45].

*Monitoring and evaluation*: Patients should be linked with dietary support services following discharge so they can be monitored and supported to achieve sustained dietary change [41].

### 4.3. Research Priorities

Large, well-designed RCTs examining dietary management of diverticulitis are a priority area for health services due to substantially increasing rates of diverticulitis and associated health care costs [6]. This review provides proof of concept that dietary intervention is able to beneficially impact upon patient outcomes in the acute and post-acute phase. For both research questions, there is a strong need to increase the certainty in the body of evidence, particularly for the high priority outcomes such as recovery, health service use, reoccurrence, patient quality of life and gastrointestinal symptoms. Future RCTs should use objective measures, blinded randomisation and allocation techniques as well as objective and/or blinded outcome assessors. Additionally, in a clinical scenario where there are multiple contributing factors to these outcomes, it is essential that potential confounders are measured and accounted for, including antibiotics use, level of inflammation, adherence, smoking, BMI and patient symptoms severity. There is emerging evidence for other risk factors having a role in the progression of the disease, such as serum vitamin D, nonsteroidal anti-inflammatory drugs, ethnicity and inherited diseases such as EDS, which also warrant further examination [46]. The large range of risk factors for diverticular disease will vary between individuals, supporting studies which used an individualized approach for management beyond standardized prescription of dietary fibre intake. Studies demonstrating the cost-effectiveness of liberalised versus restricted diets would be helpful in translation to practice, but as the “intervention” of a liberalised diet incurs no additional cost, this would not be a priority. However, identifying cost-effective methods of helping individuals to achieve long-term, sustainable high dietary fibre intakes is of much higher priority, as this will require significant redirection of nutrition resources which are already tightly strained in most health services. Alternative cost-effective options, including telehealth methods, should be investigated for appropriateness and efficacy in this population. There is a need to build evidence to determine whether a high dietary fibre diet alone is achievable and effective in improving outcomes, or whether dietary fibre supplementation should be used, and if so, the quantity and type needs further exploration. 

Finally, although probiotics may be used by patients, there is no evidence yet supporting their clinical efficacy in populations with a history of acute, uncomplicated diverticulitis. Therefore, further RCTs examining the efficacy and safety of this treatment option in addition to high dietary fibre intakes are needed. 

### 4.4. Limitations

The body of evidence is primarily limited by the absence of well conducted RCTs; but also by existing studies using varied diagnostic methods used across studies, combining intervention factors such as dietary fibre restrictions and types of antibiotics, and few using Computed Tomography for diagnosis, which is considered the gold standard diagnostic technique for acute, uncomplicated diverticulitis. This suggests the sample populations may have included patients with other similar presenting conditions such as irritable bowel syndrome, and studies may have included some patients with complicated as opposed to uncomplicated diverticulitis. As there are only a few RCTs examining both clinical research questions, this lack of evidence significantly decreased the confidence in the evidence for the treatment options, also preventing pooling of data through meta-analysis. The GRADE approach was used as the best-practice method to evaluate the body of evidence to inform treatment decisions. However, it should be noted that although the approach was used to make recommendations, this was made by the current review authors and not a robust multidisciplinary guideline panel. Additionally, it should be noted due to the very low quality of existing evidence, both clinical recommendations may change when higher quality evidence becomes available [37]. 

## 5. Conclusions

For a clinical topic of significant importance, the current body of evidence is small and of low quality for the dietary management of acute, uncomplicated diverticulitis and further research should be considered a priority area for health fundeing and service organisations internationally. However, considering the feasibility of options and values of stakeholders, recommendations for populations could be established for each clinical question. The results of this systematic review and GRADE assessment conditionally recommend the use of liberalised diets as opposed to dietary restrictions for adults with acute, uncomplicated diverticulitis. It also strongly recommends a high dietary fibre diet, which meets individual nutrient recommendations, with or without dietary fibre supplementation, after the acute episode has resolved. As this is based on available evidence which is of poor quality, recommendations may change with the availability of new higher quality evidence, which is strongly needed to better inform practice. Additionally, patients with contraindicating comorbidities or symptoms should be supported with individualised nutrition recommendations. 

## Figures and Tables

**Table 1:** Study characteristics and outcomes of studies with dietary fibre modifications in adults with acute, uncomplicated diverticulitis.

Study	Setting	Study Design	Population	Intervention	Comparator	Results
Ridgway et al. [31]	Ireland *n* = two general hospitals Data collected 2002–2004.	RCT Allocation method: randomised 1:1	Diagnosis: Acute, Uncomplicated Diverticulitis Diagnostic method: Diagnosis inferred from left iliac fossa pain and local tenderness (Hinchey ^a^ type I and II). *n* = 79 participants; 58% female μ age 66–68 years (range 31–86).	Inpatient treatment liberalised diet (“food and fluid as tolerated”) + oral abx upon admission (Ciprofloxacin 500 mg BD + Metronidazole 400 mg TDS). *n* = 41 participants	Inpatient treatment bowel rest (NBM) and IV fluids for 24 h with progression to full diet as tolerated according to daily physician consultations + oral abx introduced after 24 h (Ciprofloxacin 400 mg BD + Metronidazole 500 mg TDS). *n* = 38 participants	**Hospital LOS:** No difference between groups: liberalised diet μ5.5 (±1.9 ^b^) days vs bowel rest μ6.6 (±4.1 ^b^) days; *P* = 0.12. **Reoccurrence**: 30-day readmission rate: Acute uncomplicated diverticulitis *n* = participants (%) - liberalised diet group = 1/41 (2.4%) - bowel rest group = 1/38 (2.6%) Not compared statistically **Gastrointestinal symptoms**: Wexford Tenderness Score (score 0–4; higher score indicates higher tenderness/rigidity) at 3-days post-admission: No difference between groups: liberalised diet μ1.26 vs. bowel rest μ1.20; *P =* 0.79 **Recovery**: no treatment failures (cessation of oral therapy/crossovers) identified in either group.
Park et al. [30]	South Korea Unknown number and type of recruitment sites Data collected 2007–2009.	Prospective observational cohort study Allocation method: patient chose from treatment options	Diagnosis: Acute, right colonic uncomplicated diverticulitis Diagnostic method: Radiologic identification of inflamed diverticulum and small abscess formation *n* = 103 participants; 51% female μ age 37–40 (±10–14) years	Outpatient treatment liberalised diet + of 4-days of oral abx (second generation cephalosporins and metronidazole, with progression to ciprofloxacin monotherapy if adverse event suspected; not further specified) *n* = 40 participants	Inpatient treatment with bowel rest (nil by mouth) until symptom resolution followed by full diet (unclear if progressive stages or immediate move to full diet) + 7–10 days of IV abx (second generation cephalosporins and metronidazole, with progression to ciprofloxacin monotherapy if adverse event suspected; not further specified) *n* = 63 participants	**Hospital LOS**: μ8.1 ± 1.3 days in bowel rest + IV abx group; no comparator as outpatient group seen in outpatient clinic. **Reoccurrence**: measured up to 21 months post-diagnosis: Acute uncomplicated diverticulitis *n* = participants (%) - liberalised diet group = 4/40 (10%) - bowel rest group = 7/63 (11%) No difference between groups; *P* = 0.808. **Outpatient visits**: visits to the outpatient clinic within 1-week post-diagnosis: *n* = 2/40 in outpatient group 3-days after diagnosis. No comparator as inpatient group seen as inpatients. **Health care costs**: items included and currency not described; assumed USD: Liberalised diet and oral abx had significantly lower medical cost compared to bowel rest and IV abx (μ$1164 ± 128 vs. μ1789 ± 152; *P* < 0.001). **Recovery**: Treatment failure (no response to therapy): Liberalised diet and oral abx *n* = 2 vs. bowel rest and IV abx *n* = 0. Not compared statistically.
van de Wall et al. [12]	Netherlands Unknown number and type of recruitment sites Data collected 2010–2011	Retrospective observational cohort study Allocation method: Observation of physician treatment decisions.	Diagnosis: Acute, uncomplicated diverticulitis Diagnostic method: Modified Hinchey 0/Ia/b confirmed by CT-scan or sonography *n* = 256 participants; 57% female μ age 57–60 (±12–15) years	Inpatient liberalised diet + 26% treated with abx (not further specified) *n* = 27 participants	Inpatient bowel rest (NBM) + 40% treated with abx (not further specified). Followed by a median of 3 (range 2–4) successive inpatient diet regimens. *n* = 65 participants	**Hospital LOS** Lower in the liberalised diet compared to bowel rest (median 3 [range 2–4] days vs median 5 [range 1–16] day; not compared statistically) Liberalised diet two times more likely to be discharged compared to bowel rest in multivariate model (HR: 2.04 [95%CI: 1.27–3.29]; *P*=0.003). **Recovery**: treatment failure (development of complications including abscess, perforation or requiring surgery): *n* = 0/27 (0%) in liberalised diet, *n* = 2/65 (3.1%) in bowel rest group. Not compared statistically.
				Inpatient liberalised diet + 26% treated with abx (not further specified)	Inpatient restricted diet: clear liquids + 28% treated with abx (not further specified). Followed by a median of 3 (range 1–3) successive inpatient diet regimens. *n* = 89 participants	**Hospital LOS** Lower in the liberalised diet compared to clear liquid diet (median 3 [range 2–4] days vs median 4 [range 1–15] day; not compared statistically) **Recovery**: treatment failure (development of complications including abscess, perforation or requiring surgery): *n* = 0/27 (0%) in liberalised diet, *n* = 3/89 (3.4%) in clear liquid group. Not compared statistically.
				Inpatient liberalised diet + 26% treated with abx (not further specified)	Inpatient restricted diet: liquids + 32% treated with abx (not further specified). Followed by a median of 2 (range 1–2) successive inpatient diet regimens. *n* = 75 participants	**Hospital LOS** No difference in the liberalised diet compared to liquid diet (median 3 (range 2–4) days vs median 3 (range 1–8) day; not compared statistically) **Recovery**: treatment failure (development of complications including abscess, perforation or requiring surgery): *n* = 0/27 (0%) in liberalised diet, *n* = 1/75 (1.3%) in liquid group; not compared statistically.
Moya et al. [32]	Spain *n* = 1 general hospital Data collected 2007–2009	Historically-controlled intervention study Allocation method: consecutive admissions within defined time-period (group 1 in 2007–2008; group 2 in 2008–2009).	Diagnosis: Acute, uncomplicated diverticulitis Diagnostic method: physical examination with CT confirmation *n* = 76 participants; 53% female μ age 56–59 (range 32–84 years)	Outpatient treatment: Restricted diet (liquid only) for 4-days followed by low dietary fibre diet for 3-days with high dietary fibre diet + oral abx (Metronidazole 500 mg/8 h and Ciprofloxacin 500 mg/12 h) + IV analgesics (Acetaminophen 1 g/6 h). Patients reviewed by physician for need of hospitalisation. *n* = 32 participants	Inpatient treatment: Restricted diet (liquid only) for 3-days followed by low dietary fibre diet for 2-days with high dietary fibre diet upon discharge (5-days post diagnosis) + IV abx (Metronidazole 500 mg/8 h and Ciprofloxacin 400 mg/12 h) + IV analgesics (Acetaminophen 1 g/6 h) for 5-days followed by oral abx (Metronidazole 500 mg/8 h and Ciprofloxacin 500 mg/12 h) for 7-days *n* = 44 participants.	**Hospital LOS**: Outpatient treatment had significantly lower LOS than inpatient treatment (μ0.28 days vs. μ5.8 vs. days; *P* < 0.05). **Reoccurrence**: subsequent presentation and diagnosis within average 8–9-months post-diagnosis: Acute uncomplicated diverticulitis *n* = participants (%) - outpatient diet group = 2/34(5.9%) - inpatient diet group = 3/44(6.8%) No difference between groups; *P* = 0.86. **Health care costs**: direct health costs include ward accommodation, pharmaceutical treatment, laboratory tests and radiology: Outpatient treatment cost significantly less than inpatient treatment (μ€347.31 vs. μ€1945.26; *P* < 0.05).
Stam et al. [13]	Netherlands *n* = 1 teaching hospital Data collected 2012–2014	Prospective observational study Allocation method: One group only	Diagnosis: Acute, uncomplicated diverticulitis Diagnostic method: Modified Hinchey Ia/b *n* = 86 participants; 47% female μ age 55 (±12) years	Outpatient treatment liberalised diet (no restrictions of any kind) ^c^ + analgesics (acetaminophen or opioids if pain score over 40 on scale 0–100) + iso-osmotic laxative. Nil abx. Patients reviewed by physician for need of hospitalisation.	N/A	Outcomes assessed 6-months post-diagnosis. **Hospital admission rate**: at the time of first diagnosis: *n* = 29 (34%) admitted **Hospital LOS**: for first diagnosis: 1.8 ± 0.3 days for the 34% admitted. **Reoccurrence**: subsequent presentation and diagnosis between 3–6 months after initial diagnosis: *n* = 4 (5%) **Recovery**: treatment failure (defined by review authors as surgery, readmissions due to pain or recurrence up to 6 months post discharge) *n* = 2 (2%)

abx, antibiotics; CT, computed tomography; HR, Hazard Ratio; IV, intravenous; LOS, length of stay; NBM, nil by mouth; RCT, randomised controlled trial. ^a^ Hinchey classification is a tool used to describe successive stages of perforations (severity) of diverticulitis [33]; ^b^ This study reported standard errors; however, we have reported standard deviations, calculated by Review Manager; ^c^ Not all patients with acute, uncomplicated diverticulitis were given liberalised diet and recruited: there were *n* = 70 patients excluded from participating due patient-reported inability to tolerate any oral intake; need for antibiotics, immunocompromised; declined participation; and suspicion of inflammatory bowel disease or malignancy.

**Table 2:** Study characteristics and outcomes of studies that compare dietary modifications to increase dietary fibre for the management of uncomplicated diverticulitis after the acute episode has resolved.

Study	Setting	Study Design	Population	Intervention	Comparator	Results
Taylor and Duthie [35]	UK Unknown number and type of recruitment sites Data collected: dates not specified	Three-arm randomised cross-over intervention study Allocation method: random allocation (not further described).	Diagnosis: symptomatic diverticular disease; 40% with recent acute, uncomplicated diverticulitis Diagnostic method: barium enema. *n* = 20 participants. Gender and age not reported.	One month of high fibre diet (termed high-roughage diet) with 18 g dietary fibre from supplements (9 × 2 g bran tables per day). Written educational material provided for high-roughage diet.	One month of dietary fibre supplement with laxative (Normacol: sterculia with frangula bark—dosage not specified) with anti-spasmodic	**Gastrointestinal symptoms**: at one-month post intervention: gastrointestinal symptom scores (scale 0–17; higher score indicating worse symptoms): Score reported to improve in both groups (data not provided). Ongoing gastrointestinal symptoms High fibre diet + supplements had more participants with ongoing symptoms compared to laxative group (*n* = 10/13 [80%] vs. *n* = 8/13 [60%]). Not compared statistically. Stool weight: Increased in both groups but was not statistically significant different between groups (high fibre diet + supplements μ102 g ± S.E: 15.9 vs. normacol μ105 ± 13.5). Transit time: Decreased significantly in all groups but no difference between groups (high fibre diet + supplements μ76.4 ± S.E:7.2 h vs. normacol μ71.7 ± 10.9 h).
				One month of 18g dietary fibre from supplements (9 × 2 g bran tables per day) with no education regarding dietary change.	One month of dietary fibre supplement with laxative (Normacol: sterculia with frangula bark—dosage not specified) with anti-spasmodic	**Gastrointestinal symptoms**: at one-month post intervention: gastrointestinal symptom scores (scale 0–17; higher score indicating worse symptoms): Score reported to improve in both groups (data not provided). Ongoing gastrointestinal symptoms Bran supplements had fewer participants with ongoing symptoms compared to normacol group (*n* = 5/13 [40%] vs. *n* = 8/13 [60%]). Not compared statistically. Stool weight: Stool weight statistically increased in both groups, and bran supplement was statistically more effective in increasing stool weight compared to the normacol (μ121 g ± S.E:7.1 vs. μ105 ± 13.5) Transit time: Decreased significantly in all groups, and bran supplement was statistically more effective in decreasing transit time compared to normacol (μ56.1 ± S.E:4.1 h vs. μ71.7 ± 10.9 h; *P* < 0.05).
				One month of high-roughage diet with 18 g dietary fibre from supplements (9 × 2 g bran tables per day). Written educational material provided for high-roughage diet.	One month of 18 g dietary fibre from supplements (9 × 2 g bran tables per day) with no education regarding dietary change.	**Gastrointestinal symptoms**: at one-month post intervention: gastrointestinal symptom scores (scale 0–17; higher score indicating worse symptoms): Score reported to improve in both groups (data not provided). Ongoing gastrointestinal symptoms Bran supplements had fewer participants with ongoing symptoms compared to high roughage diet + supplements (*n* = 5/13 [40%] vs. *n* = 10/13 [80%]). Not compared statistically. Stool weight: Stool weight statistically increased in both groups, and bran was statistically more effective in increasing stool weight compared to the high fibre and supplement group (μ102 g ± S.E: 15.9 vs. μ102 g ± S.E: 15.9) Transit time: Decreased significantly in all groups, and bran supplement was statistically more effective in decreasing transit time compared to high fibre diet + supplement (μ56.1 ± S.E:4.1 h vs. μ76.4 ± S.E: 7.2 h; *P* < 0.001).
Leahy et al. [36]	UK *n* = 1 general hospital Data collected: 1972–1981	Retrospective observational cohort study Allocation method: 76% received high fibre education during hospitalisation. Others did not receive for unreported reason.	Diagnosis: symptomatic, uncomplicated Diverticulitis requiring hospitalisation (acute) Diagnostic method: barium enema or radiological examination + symptoms *n* = 56 participants. Gender and age not reported.	Adhering to a high fibre diet (≥25 g dietary fibre/day with or without dietary fibre supplements) 2–11 years after initial hospitalisation. Education was initially given during hospital admission patient counselled by medical and dietetic staff with written educational material. Other in-hospital treatments not described.	Low fibre diet (<25 g/day) allocated by not adhering to high fibre diet recommended in hospital or were not educated regarding high fibre diet).	**Reoccurrence**: readmission rate at 54–76 months post-diagnosis: Fewer reoccurrence of acute episode in high fibre diet group than low fibre diet group (*n* = 2/31 [7%] vs. *n* = 5/25 [20%]; *P* < 0.05). **Gastrointestinal symptoms**: patient reported ongoing symptoms (dichotomous, no scale/tool used) at 54–76 months post-diagnosis: High fibre group had fewer ongoing symptoms compared to low fibre group (*n* = 6/22 [27%] vs. *n* = 11/16 [69%]; *P* < 0.05).
Lanas et al. [34]	Italy Multicentre: 23 gastroentero-logical centres. Data collected: 2007–2008	RCT Allocation method: computer generated random allocation	Diagnosis: acute diverticulitis (94% uncomplicated), within 2 months prior to recruitment Diagnostic method: confirmed by CT scan, ultrasonography or endoscopy *n* = 165 participants; 37% female. μ 54–55 years (±12–13).	7 g dietary fibre supplementation (3.5 g plantago ovata husk [psyllium]] consumed as effervescent granulate BD) consumed daily for 48 weeks. Dietary fibre consumed from diet not measured.	7 g dietary fibre supplementation (3.5 g plantago ovata husk [psyllium]] consumed as effervescent granulate BD) consumed daily for 48 weeks + poorly absorbed oral abx (400 mg rifaximin polymorph alpha BD) consumed for one week of each month for 48 week. Dietary fibre consumed from diet not measured.	**Reoccurrence**: readmission rate at 48 weeks from baseline: Fewer reoccurrence of acute episode in supplement + abx group than supplement alone group (*n* = 8/77 [10%] vs. *n* = 17/88 [19%]), compared statistically in multivariate model (HR of reoccurrence in supplement only group: 2.64 [95%CI: 1.08–6.46; *P* = 0.033). **Gastrointestinal symptoms**: unknown score (scored 0–10, 10 worse symptoms; using visual analogue scale and number of diarrhoea episodes) reflecting gastrointestinal symptoms at 48 weeks from baseline: No improvement from baseline for both groups or difference between groups at follow-up (supplement + abx μ3.45 ± 7.03 vs supplement only group μ3.26 ± 5.81); not compared statistically.

abx, antibiotics; CT, computed tomography; g, grams; h, hours; RCT, randomised controlled trial; S.E., standard error.

## Supplementary Materials

The following are available online at http://www.mdpi.com/2072-6643/10/2/137/s1, Online Supplementary Material 1: Search Strategy; Online Supplementary Material 2: Cochrane Risk of Bias Assessment; Online Supplementary Material 3: GRADE recommendation.
