# Supplementary material for: Evidence for Dietary Fibre Modification in the Recovery and Prevention of Reoccurrence of Acute, Uncomplicated Diverticulitis: A Systematic Literature Review
Source: Nutrients. 2018 Jan 27;10(2):137. doi: 10.3390/nu10020137 (PMC5852713; doi:10.3390/nu10020137)
Supplement: Supplementary file 1 [file nutrients-10-00137-s001.docx]

**Online Supplementary Material 3: GRADE Assessments for each clinical research question used to inform recommendations**

| Question | |
| --- | --- |
| Should a **liberalised diet** vs. **restricted diet** be used for the **dietary management of acute, uncomplicated diverticulitis**? | |
| **Population:** | Dietary management of acute, uncomplicated diverticulitis |
| **Intervention:** | Liberalised diet |
| **Comparison:** | Restricted diet |
| **Main outcomes:** | Hospital length of stay; Reoccurrence; Recovery time; Gastrointestinal symptoms; Reoccurrence length of stay; Health service costs; Patient satisfaction; Quality of life; Number of Bowel openings; Diarrhoea; Constipation; Stool consistency and frequency; Stool weight; Abdominal Pain. |
| **Setting:** | Any health care setting |
| **Perspective:** | Treating clinical team |

## Evidence table

| **Quality assessment** | | | | | | | **№ of patients** | | **Effect** | | **Quality** | **Importance** |
| --- | --- | --- | --- | --- | --- | --- | --- | --- | --- | --- | --- | --- |
| **№ of studies** | **Study design** | **Risk of bias** | **Inconsistency** | **Indirectness** | **Imprecision** | **Other considerations** | **liberalised diet** | **restricted diet** | **Relative (95% CI)** | **Absolute (95% CI)** |  |  |
| Hospital length of stay (follow up: range 1 days to 16 days; assessed with: Days of initial admission; Scale from: 1 to 100) | | | | | | | | | | | | |
| 5 ^a^ | observational studies ^b^ | serious ^c^ | not serious | serious ^d^ | not serious | none | 162 | 330 | not estimable | not estimable | ⨁◯◯◯ VERY LOW | CRITICAL^j^ |
| Reoccurrence (follow up: range 1 days to 21 months; assessed with: Incidence/rate of subsequent admission to hospital with primary diagnosis of diverticulitis) | | | | | | | | | | | | |
| 2 | observational studies ^e^ | serious ^c^ | not serious | serious ^d^ | serious ^f^ | none | 81 | 93 | not estimable | not estimable | ⨁◯◯◯ VERY LOW | CRITICAL^k^ |
| Recovery (follow up: range 1 weeks to 3 weeks; assessed with: Rate of treatment failures) | | | | | | | | | | | | |
| 5 ^a^ | observational studies ^b^ | serious ^c^ | not serious | not serious | serious | none | 168 | 330 | not estimable | not estimable | ⨁◯◯◯ VERY LOW | CRITICAL^l^ |
| Gastrointestinal symptoms (follow up: 5 years; assessed with: Scale of reported gastrointestinal symptoms [Wexford Tenderness Score; Scale from: 0 to 4]) | | | | | | | | | | | | |
| 1 | randomised trials | serious ^c^ | not serious ^g^ | serious ^d^ | very serious ^h^ | none | 41 | 38 | not estimable | not estimable | ⨁◯◯◯ VERY LOW | IMPORTANT |

**CI:** Confidence interval

a. Although Moya et. al. and Stam et. al. reported this outcome, they did not have comparisons for liberalised versus restricted diet and therefore were not included. Van der wall had three groups with three comparisons, and therefore was considered as three studies.

b. There were a range of study designs, including a RCT, a prospective observational study and a retrospective observational study

c. Risk of bias informed by the Cochrane risk of bias tool. Results in Figure 2.

d. Poor generalisability due to selection bias and small number and sample size of studies; and significant clinical heterogeneity with the types of treatments provided and/or characteristics such as dietary intake poorly described.

e. The two study designs for this outcome were RCT and prospective observational study

f. Some imprecision as expected when sample size is less than 300-400. The confidence intervals for this outcome were somewhat large, though not highly concerning.

g. As only one study measured this, this could not really be assessed

h. Only one study measured this outcome with a tool which has not been vigorously validated; Therefore, imprecision is likely.

# Assessment

|  | **Judgement** | **Research evidence** | **References** |
| --- | --- | --- | --- |
| Problem | **Is the problem a priority?**  ○ No ○ Probably no ○ Probably yes ● Yes ○ Varies ○ Don't know | The incidence, prevalence, and number of hospital admissions are rapidly increasing due to a rising prevalence of risk factors in the population such as ageing, increasing adiposity and sedentary lifestyles, and low diet quality (Böhm 2015; Vather et. al. 2015; Aune et. al. 2017). The estimated annual 300,000 acute diverticulitis admissions in the United States of America has a financial burden of USD$2.5billion (£1.9billion). Upon diagnosis of acute uncomplicated diverticulitis (i.e. not requiring surgical intervention), short-term low dietary fibre intake or food deprivation for bowel rest, with or without the administration of antibiotics, is often used in clinical practice as it is thought that a less active bowel reduces colonic irritation and re-inflammation (de Korte et. al. 2011; Rafferty et. al. 2006). These treatment options are usually administered in the inpatient setting, but may also be provided as outpatient care. However, the efficacy and safety of these treatment approaches has not yet been reviewed, and a restricted diet may be associated with longer hospital stay, increased patient burden, increased risk of malnutrition especially in older adults, and increased health care costs. Due to the lack of evidence regarding dietary restriction during inpatient treatment, practice varies widely from physician to physician, and evidence guidelines provide minimal and conflicting recommendations. | Aune, D., et al., *Body mass index and physical activity and the risk of diverticular disease: a systematic review and meta-analysis of prospective studies.* European Journal of Nutrition, 2017: p. 1-16.  Böhm, S.K., *Risk Factors for Diverticulosis, Diverticulitis, Diverticular Perforation, and Bleeding: A Plea for More Subtle History Taking.* Visceral Medicine, 2015. **31**(2): p. 84-94.  de Korte, N., et al., *Management of diverticulitis: results of a survey among gastroenterologists and surgeons.* Colorectal Dis, 2011. **13**(12): p. e411-7.  Rafferty, J., et al., *Practice parameters for sigmoid diverticulitis.* Dis Colon Rectum, 2006. **49**(7): p. 939-44.  Vather, R., et al., *Demographics and trends in the acute presentation of diverticular disease: a national study.* ANZ J Surg, 2015. **85**(10): p. 744-8. |
| Desirable Effects | **How substantial are the desirable anticipated effects?**  ○ Trivial ○ Small ● Moderate ○ Large ○ Varies ○ Don't know | The anticipated effect of a likely decrease in length of stay is assumed by the authors to be highly desirable by both patients and health care providers. Due to the high cost of hospitalisation per day, the authors have assumed that even small effect sizes in decreasing length of stay for patients with diverticulitis will decrease health care cost, as is the case with other clinical conditions, such as pneumonia and post colon surgery (Fine et. al. 2000; Stephen & Berger, 2003).  Likelihood of other anticipated effects are equal between the two diet types, and are also assumed to be highly desirable. These include patient recovery (low risk of treatment failure), low risk of reoccurrence and no exacerbated gastrointestinal symptoms. Most studies were observational; however, the RCT which randomly allocated 80 patients to bowel rest or a liberalised diet identified no complications or treatment failures; therefore, liberalised diets do appear to be safe. It is acknowledged though that this is a small study and larger studies would help to build the evidence regarding the safety of liberalised diets.  As there is no evidence of difference between liberalised diet and restricted diet in terms of patient recovery (treatment failure), reoccurrence and gastrointestinal symptoms, there is no expected undesirable anticipated effects. However, some patients (and care givers) may experience confusion if they have previously been placed on bowel rest if previously admitted for diverticulitis, and surgeons may be concerned to go against some expert opinion-based guidelines which recommend bowel rest. | Fine, M. J., Pratt, H. M., Obrosky, D. S., et al. 2000. Relation between length of hospital stay and costs of care for patients with community-acquired pneumonia. *The American journal of medicine,* 109**,** 378-385.  Stephen, A. E. & Berger, D. L. 2003. Shortened length of stay and hospital cost reduction with implementation of an accelerated clinical care pathway after elective colon resection. *Surgery,* 133**,** 277-282. |
| Undesirable Effects | **How substantial are the undesirable anticipated effects?**  ○ Large ○ Moderate ● Small ○ Trivial ○ Varies ○ Don't know |  |  |
| Certainty of evidence | **What is the overall certainty of the evidence of effects?**  ● Very low ○ Low ○ Moderate ○ High ○ No included studies | The overall certainty in the body of evidence was very low; however, this was for both liberalised and restricted diets. The evidence that does exist, although low quality, shows no difference between the two diets, excepting that a liberalised diet may be associated with decreased length of stay. With this in mind, there is no evidence of effect in existing literature to support withholding food or restricting diets as opposed to providing a liberalised diet, which is consistent also for the dietary management of most other conditions requiring hospital admission. |  |
| Values | **Is there important uncertainty about or variability in how much people value the main outcomes?**  ○ Important uncertainty or variability ○ Possibly important uncertainty or variability ○ Probably no important uncertainty or variability ● No important uncertainty or variability | The main outcomes were hospital length of stay, recovery (treatment failures/complications), reoccurrence, patient symptoms and health care costs. Although there are no qualitative studies examining patient and health care provider opinions on the importance of these outcomes, they directly reflect the severity and recovery of a medical condition with high patient and health care burden, the authors have assumed there is no uncertainty about the value of these outcomes. |  |
| Balance of effects | **Does the balance between desirable and undesirable effects favour the intervention or the comparison?**  ○ Favours the comparison ○ Probably favours the comparison ○ Does not favour either the intervention or the comparison ● Probably favours the intervention ○ Favours the intervention ○ Varies ○ Don't know | The evidence appears to favour the intervention, as there are currently no undesirable health outcomes identified. However, it must be acknowledged that the quality of studies available are limited, and therefore confidence in this is limited.  The undesirable effects identified by authors relate mainly to preferences of the patient if they have received a bowel rest diet with previous admissions and possible resistance from some health care providers who usually recommend bowel rest. Regarding patient preferences, if a patient does not wish to eat, then the individual may refuse food or not consume provided food at their own discretion. Conversely, not allowing the patient that choice is assumed to be against the general patients’ wishes. Therefore, this concern is easily addressed.  Some health care providers may resist using liberalised diets if they do not usually use this approach, and/or because some expert-opinion papers still recommend bowel rest. However, it is assumed by the authors that providers do not wish to decrease food/nutrient intake when there is no supporting intervention evidence to do so. In this case, if a health care provider has concerns about the safety of a liberalised diet for a patient, they are still encouraged to restrict the diet on an individualised basis.  Overall, the authors believe desirable effects of a liberalised diet outweigh patient and health care provider resistance. |  |
| Resources required | **How large are the resource requirements (costs)?**  ○ Large costs ○ Moderate costs ○ Negligible costs and savings ● Moderate savings ○ Large savings ○ Varies ○ Don't know | It is not ethical to withhold food and place dietary restrictions as a measure of savings on the food budget, therefore cost-savings associated with dietary restrictions are not considered by the authors. As a liberalised diet in fact places "no intervention", there is no cost/resource required. The evidence suggests that a liberalised diet is associated with decreased length of stay, and therefore may result in savings in health care costs. There is no economic analysis available for this clinical question; however, decreases in length of stay have been associated with significant cost savings for other conditions (Fine et. al. 2000; Stephen & Berger, 2003) and it is expected that decreases in diverticulitis-related admissions will have similar savings. | Fine, M. J., Pratt, H. M., Obrosky, D. S., et al. 2000. Relation between length of hospital stay and costs of care for patients with community-acquired pneumonia. *The American journal of medicine,* 109**,** 378-385.  Stephen, A. E. & Berger, D. L. 2003. Shortened length of stay and hospital cost reduction with implementation of an accelerated clinical care pathway after elective colon resection. *Surgery,* 133**,** 277-282. |
| Certainty of evidence of required resources | **What is the certainty of the evidence of resource requirements (costs)?**  ○ Very low ○ Low ○ Moderate ○ High ● No included studies | Although there is no study confirming that the implementation of a liberalised diet has no cost, this is due to the simple understanding that not imposing a restriction allows food and beverage to be given to patients as per usual care. There are no additional steps by health care providers in allowing for a liberalised diet, and therefore, even in the lack of studies, the authors are very confident about the negligible resources/costs associated with liberalised diets.  On the other hand, we are uncertain about the costs of implementing a restricted diet, but agree they are likely to be higher. This is due to health care providers needing to review patients for dietary changes (progression towards a full diet), document these and then have other staff involved in implementing them (e.g. nursing and/or dietetics). |  |
| Cost effectiveness | **Does the cost-effectiveness of the intervention favour the intervention or the comparison?**  ○ Favours the comparison ○ Probably favours the comparison ○ Does not favour either the intervention or the comparison ○ Probably favours the intervention ○ Favours the intervention ○ Varies ● No included studies | No cost-effectiveness studies have been done; however, it is evident that "no intervention" in terms of not restricting diets imposes no cost. If the two treatments are equally clinically effective, then the no cost option (liberalised diet) which also increases nutrient intake and choice can be assumed to be the more cost-effective option. |  |
| Equity | **What would be the impact on health equity?**  ○ Reduced ○ Probably reduced ○ Probably no impact ● Probably increased ○ Increased ○ Varies ○ Don't know | There are no sub-groups recognised by the authors which would be positively or negatively affected by choosing either option. However, increased patient choice and equal access to food is more likely to meet consumer expectations and improve determinants of health, particularly for at-risk groups, such as older adults, those with increased nutrient requirements or with food insecurity. |  |
| Acceptability | **Is the intervention acceptable to key stakeholders?**  ○ No ○ Probably no ● Probably yes ○ Yes ○ Varies ○ Don't know | Qualitative studies have not been conducted; however, allowing for patient choice in consuming offered foods and beverages is assumed to be acceptable to patients. If there is no evidence-based clinical indication for dietary restriction, then it is assumed this will be acceptable to health care providers and health services. However, the authors recognise that health care providers may wish to be cautious as dietary restrictions and bowel rest have been traditionally used worldwide. Therefore, the authors accept there may be some resistance from some health care providers. |  |
| Feasibility | **Is the intervention feasible to implement?**  ○ No ○ Probably no ○ Probably yes ● Yes ○ Varies ○ Don't know | Providing a liberalised diet is no cost, and is likely to require less management by health care providers. It allows for usual/standard hospital inpatient care and therefore is immediately feasible with no changes required to any health service or food service systems. |  |

## Recommendation:

*Recommendation for the population*: Adult patients admitted to hospital with acute, uncomplicated diverticulitis (i.e. no perforation, abscess, drains placed or surgery required) should be placed on a liberalised diet (i.e. allowing consumption of solid food) and not placed on a restricted diet (i.e. bowel rest/nil by mouth, clear and/or liquid diets).

*Strength of the recommendation:* Strong recommendation for the intervention (liberalised diets) based on a very low-quality body of evidence.

*Overall Justification:* This review identified no evidence of a difference between liberalised and restricted diets in terms of clinical outcomes including recovery (treatment failures), reoccurrence or patient symptoms; however, liberalised diets may decrease length of hospital stay and prevent restriction of essential nutrient intake (e.g. dietary fibre, vitamins, minerals, phytonutrients, energy and protein found in solid foods) in patients. It can be generally accepted that the majority of patients would prefer autonomy and/or not to have food restrictions (such as nil by mouth or liquid only diets) prescribed unless there is evidence of a contraindication. It can also be generally accepted that health care providers would rather not place further nutrition restrictions on patients, which may require increased dietary management by physicians, dietitians and/or nursing staff, when unsupported by the evidence. Placing patients on a liberalised diet is highly feasible. Therefore, this recommendation was considered strong based on a low quality of evidence, as using a liberalised diet has a low risk and benefits clearly outweigh burden of implementing a restricted diet [27]. This type of recommendation aligns with recommendations made by the American Society of Colon and Rectal Surgeons (ASCRS) for the medical management of acute diverticulitis [17]. However, health care providers should consider the individual risk profiles of patients to identify other potential contraindications for oral intake, such as co-morbidities or risk for the development of complications (i.e. presence of bleeding, abscess or perforation).

*Detailed Justification:*

- *Values*: It is generally accepted that patients and health care providers do not wish to impose unnecessary dietary restrictions, which may cause patient and health care provider burden, decreased nutrient intake, and increased risk of malnutrition for older adults. It is generally accepted that decreased length of hospital stay is desirable for patients, health care providers and the health service.
- *Resources required*: There are no resources required to implement the intervention; and there is a likely to be a saving of resources (staff time) by not implementing a restricted diet, which requires ongoing monitoring and adjustments.
- *Cost effectiveness:* There is no cost to implement the intervention, but likely increased costs to implement the comparison (restricted diet) through increased health care provider management of dietary restrictions, and/or increased risk of complications associated with nutrient/food deprivation (e.g. malnutrition).
- *Acceptability*: A liberalised diet is likely to be acceptable to patients and health care providers. Liberalised diets are not forcing the patient to eat if they do not wish to; it is simply offering the freedom of choice to consume food. It is likely to require less health care resources and impose less burden on patients.

*Subgroup considerations:* Restriction of dietary intake in older adults (≥65 years), patients with increased nutrient requirements, and those with food insecurity (e.g. financial insecurity or drug/alcohol-dependence) should be minimised as much as possible due to their significantly higher risk of malnutrition.

*Implementation considerations:* There are minimal implementation considerations. Implementation of the intervention is simply not imposing any dietary restrictions.

*Monitoring and evaluation:* Patients should be monitored closely, as per current recommendations, for signs of worsening condition and/or progression to complicated diverticulitis.

## Summary of judgements

|  | **Judgement** | | | | | | | **Implications** |
| --- | --- | --- | --- | --- | --- | --- | --- | --- |
| **Problem** | No | Probably no | Probably yes | **Yes** |  | Varies | Don't know | Favours liberalised diet |
| **Desirable Effects** | Trivial | Small | **Moderate** | Large |  | Varies | Don't know | Probably favours liberalised diet |
| **Undesirable Effects** | Large | Moderate | **Small** | Trivial |  | Varies | Don't know | Probably favours liberalised diet |
| **Certainty of evidence** | **Very low** | Low | Moderate | High |  |  | No included studies | Probably favours liberalised diet |
| **Values** | Important uncertainty or variability | Possibly important uncertainty or variability | Probably no important uncertainty or variability | **No important uncertainty or variability** |  |  |  | Probably favours liberalised diet |
| **Balance of effects** | Favours the comparison | Probably favours the comparison | Does not favour either the intervention or the comparison | **Probably favours the intervention** | Favours the intervention | Varies | Don't know | Probably favours liberalised diet |
| **Resources required** | Large costs | Moderate costs | Negligible costs and savings | **Moderate savings** | Large savings | Varies | Don't know | Favours liberalised diet |
| **Certainty of evidence of required resources** | Very low | Low | Moderate | High |  |  | **No included studies** | Probably favours liberalised diet |
| **Cost effectiveness** | Favours the comparison | Probably favours the comparison | Does not favour either the intervention or the comparison | Probably favours the intervention | Favours the intervention | Varies | **No included studies** | Probably favours the intervention |
| **Equity** | Reduced | Probably reduced | Probably no impact | **Probably increased** | Increased | Varies | Don't know | Probably favours the intervention |
| **Acceptability** | No | Probably no | **Probably yes** | Yes |  | Varies | Don't know | Probably favours liberalised diet |
| **Feasibility** | No | Probably no | Probably yes | **Yes** |  | Varies | Don't know | Favours liberalised diet |

| Question | |
| --- | --- |
| Should a **high dietary fibre diet** vs. **standard or low fibre diet** be used for the **management of acute diverticulitis after the initial episode has resolved**? | |
| **Population:** | Prevention of acute diverticulitis reoccurrence |
| **Intervention:** | High dietary fibre diet |
| **Comparison:** | Standard or low fibre diet |
| **Main outcomes:** | Reoccurrence; Gastrointestinal symptoms; Bowel transit time; Rehospitalisation length of stay; Health care costs; Patient satisfaction; Quality of life; Bowel openings; Diarrhoea; Constipation; Stool consistency; Stool weight; Pain; Outpatient medical visits regarding gastrointestinal concerns; |
| **Setting:** | Post-hospital discharge / outpatient / community |
| **Perspective:** | Treating clinical team |

## Evidence Table

| **Quality assessment** | | | | | | | **№ of patients** | | **Effect** | | **Quality** | **Importance** |
| --- | --- | --- | --- | --- | --- | --- | --- | --- | --- | --- | --- | --- |
| **№ of studies** | **Study design** | **Risk of bias** | **Inconsistency** | **Indirectness** | **Imprecision** | **Other considerations** | **high dietary fibre diet** | **standard or low fibre diet** | **Relative (95% CI)** | **Absolute (95% CI)** |  |  |
| Reoccurrence (follow up: range 4.5 years to 6.3 years; assessed with: Rate of hospital readmissions where the primary diagnosis is diverticulitis) | | | | | | | | | | | | |
| 1 | observational studies | very serious ^a^ | not serious | not serious | very serious ^b^ | strong association | 31 | 25 | not estimable | not estimable | ⨁◯◯◯ VERY LOW | CRITICAL ^c^ |
| Gastrointestinal symptoms (follow up: range 4.5 years to 6.3 years; assessed with: Reporting of ongoing GI symptoms at follow-up) | | | | | | | | | | | | |
| 1 | observational studies | very serious ^a^ | not serious | not serious | very serious ^b^ | very strong association | 22 | 16 | not estimable | not estimable | ⨁◯◯◯ VERY LOW | IMPORTANT ^d^ |

**CI:** Confidence interval

a. Risk of bias informed by the Cochrane risk of bias tool. Results in Figure 2.

b. Low confidence in precision due to only one study with very low patient numbers.

c. Prevention of another acute episode of diverticulitis is the primary clinical goal following resolution of the initial episode, from both patient and health service perspective.

d. Ongoing patient gastrointestinal symptoms may present a detrimental impact on the patients’ long-term quality of life

# Assessment

|  | **Judgement** | **Research evidence** | **References** |
| --- | --- | --- | --- |
| Problem | **Is the problem a priority?**  ○ No ○ Probably no ● Probably yes ○ Yes ○ Varies ○ Don't know | Following resolution of acute symptoms, patients are frequently recommended to follow a low dietary fibre diet for a short period of time and then transition to a high dietary fibre diet (Feingold et. al. 2014; NEMO). A high dietary fibre diet is hypothesised to prevent diverticulitis reoccurrence due to its potential for more frequent bowel movements, thus reducing the contact time between gut contents and diverticula, and the associated irritation that can lead to diverticulitis (Commane et. al 2009; Tursi, 2007). However, despite frequent recommendations of high dietary fibre diets, with or without dietary fibre supplements, there has been no systematic review and critical appraisal of the evidence of these approaches to prevent acute diverticulitis reoccurrence. This is reflected by inconsistent guidelines which are based on low quality evidence (Peery et. al. 2012; Strate, 2012) and is likely to have important clinical implications, including patient burden and increased health service use. | Commane, D.M., et al., *Diet, ageing and genetic factors in the pathogenesis of diverticular disease.* World J Gastroenterol, 2009. **15**(20): p. 2479-88.  Feingold, D., et al., *Practice parameters for the treatment of sigmoid diverticulitis.* Diseases of the Colon and Rectum, 2014. **57**(3): p. 284-294.  Peery, A.F., et al., *A high-fiber diet does not protect against asymptomatic diverticulosis.* Gastroenterology, 2012. **142**(2): p. 266-72.e1.  Strate, L.L., *Diverticulosis and dietary fiber: rethinking the relationship.* Gastroenterology, 2012. **142**(2): p. 205-7.  Tursi, A., *New physiopathological and therapeutic approaches to diverticular disease of the colon.* Expert Opin Pharmacother, 2007. **8**(3): p. 299-307  *Queensland Health Nutrition Education Materials Online (NEMO)*. Available from: <https://www.health.qld.gov.au/nutrition>. |
| Desirable Effects | **How substantial are the desirable anticipated effects?**  ○ Trivial ○ Small ○ Moderate ● Large ○ Varies ○ Don't know | One study, despite being small, of low-quality and the only one of its kind, still produced a very large effect size on the rate of reoccurrence and the decrease in patient symptoms. This is underpinned by a strong theoretical background supporting the beneficial effects of a high fibre diet (Commane et. al 2009; Tursi,2007), not only for improvement in outcomes for acute, uncomplicated diverticulitis, but for overall health as per several national dietary guidelines (Australian Dietary Guidelines, 2013; The EatWell Guide, 2016). As consuming a high dietary fibre diet is recommended by national guidelines, there are no undesirable effects from a health perspective, excepting perhaps short term gastrointestinal symptoms experienced by some people with intolerances (Staudacher, et. al. 2011). However, if patients participate in dietary or nutrition programs to support dietary change this may pose some cost to patients. | *Australian Dietary Guidelines*. 2013, NHMRC, Australian Government: Canberra.  Choices, N., *The Eatwell Guide*. 2016.  Commane, D.M., et al., *Diet, ageing and genetic factors in the pathogenesis of diverticular disease.* World J Gastroenterol, 2009. **15**(20): p. 2479-88.  Staudacher, H. M., Whelan, K., Irving, P. M., et al. 2011. Comparison of symptom response following advice for a diet low in fermentable carbohydrates (fodmaps) versus standard dietary advice in patients with irritable bowel syndrome. *J. Hum. Nutr. Diet.,* 24**,** 487-495. Tursi, A., *New physiopathological and therapeutic approaches to diverticular disease of the colon.* Expert Opin Pharmacother, 2007. **8**(3): p. 299-307 |
| Undesirable Effects | **How substantial are the undesirable anticipated effects?**  ○ Large ○ Moderate ● Small ○ Trivial ○ Varies ○ Don't know |  |  |
| Certainty of evidence | **What is the overall certainty of the evidence of effects?**  ● Very low ○ Low ○ Moderate ○ High ○ No included studies | The certainty of the evidence for the improvement in outcomes related to diverticulitis is very low, as the two groups have been evaluated by only one small observational study. However, there is strong evidence supporting a high dietary fibre intake for overall health, as well as other specific chronic diseases (NHMRC, 2005). | Nutrient Reference Values for Australia and New Zealand Including Recommended Dietary Intakes. 2005, National Health and Medical Resarch Council: Canberra. |
| Values | **Is there important uncertainty about or variability in how much people value the main outcomes?**  ○ Important uncertainty or variability ○ Possibly important uncertainty or variability ○ Probably no important uncertainty or variability ● No important uncertainty or variability | It is assumed by the authors that all patients, following acute, uncomplicated diverticulitis, desire to prevent diverticulitis from occurring again and wish to avoid ongoing gastrointestinal symptoms. There is strong certainty that this is the goal of health care providers, as many hospital discharge services provide routine education for a high fibre diet even in the absence of high quality evidence to support the recommendation. Interventions to prevent reoccurrence are also addressed by clinical guidelines (Wilkins et. al. 2013). | Wilkins, T., Embry, K. & George, R. 2013. Diagnosis and management of acute diverticulitis. *Am. Fam. Physician,* 87. |
| Balance of effects | **Does the balance between desirable and undesirable effects favour the intervention or the comparison?**  ○ Favours the comparison ○ Probably favours the comparison ○ Does not favour either the intervention or the comparison ● Probably favours the intervention ○ Favours the intervention ○ Varies ○ Don't know | Undesirable effects have been described as temporary gastrointestinal discomfort as increases in dietary fibre intake are made; however, these effects are temporary (Cummings, 1973). There is good evidence to show that long term changes to dietary patterns and habits are hard to make for the general population, and therefore patients are likely to need support (Pinho et. al. 2017). It should also be acknowledged not all people desire to make dietary change, even if there is good evidence of improved outcomes (Marshall et. al. 2017). Overall, there is still greater gain in achieving a high dietary fibre diet due to the improvement in overall health (Australian Dietary Guidelines, 2013; The EatWell Guide, 2016), as well as potential for the prevention of diverticulitis reoccurrence and reduction of symptoms. | *Australian Dietary Guidelines*. 2013, NHMRC, Australian Government: Canberra.  Choices, N., *The Eatwell Guide*. 2016.  Cummings, J. 1973. Dietary fibre. Gut, 14**,** 69-81.  Marshall, S., Reidlinger, D. P., Young, A., et al. 2017. The nutrition and food-related roles, experiences and support needs of female family carers of malnourished older rehabilitation patients. *J. Hum. Nutr. Diet.,* 30**,** 16-26.  Pinho, M., et al., Exploring the relationship between perceived barriers to healthy eating and dietary behaviours in European adults. European journal of nutrition, 2017: p. 1-10. |
| Resources required | **How large are the resource requirements (costs)?**  ○ Large costs ● Moderate costs ○ Negligible costs and savings ○ Moderate savings ○ Large savings ○ Varies ○ Don't know | Sustained dietary change is hard to achieve for most people, as the general food supply does not promote a high fibre diet (Roberto et. al 2015). Therefore, ongoing support, such as that from dietitians or commercial programs is required to assist individuals in implementing dietary change. These can be resource intensive, and cost/QALY should be examined for methods of dietary intervention, as this may find cost-effectiveness of some types of intervention are higher than others, e.g. telehealth vs online programs vs one-on-one dietary counselling (Free et. al. 2013; Gordon et. al 2007). However, achieving a high dietary fibre diet aligns with dietary guidelines and therefore is still recommended even in the absence of cost-effectiveness data (Australian Dietary Guidelines, 2013; The EatWell Guide, 2016). | *Australian Dietary Guidelines*. 2013, NHMRC, Australian Government: Canberra.  Choices, N., *The Eatwell Guide*. 2016.  Free, C., et al., The effectiveness of mobile-health technology-based health behaviour change or disease management interventions for health care consumers: a systematic review. PLoS med, 2013. **10**(1): p. e1001362  Gordon, L., et al., A review of the cost-effectiveness of face-to-face behavioural interventions for smoking, physical activity, diet and alcohol. Chronic Illness, 2007. **3**(2): p. 101-129.  Roberto, C.A., et al., Patchy progress on obesity prevention: emerging examples, entrenched barriers, and new thinking. The Lancet, 2015. **385**(9985): p. 2400-2409 |
| Certainty of evidence of required resources | **What is the certainty of the evidence of resource requirements (costs)?**  ○ Very low ○ Low ○ Moderate ○ High ● No included studies | This study did not find any evidence regarding resource requirements. There are a high variety of options available with which dietary change can be supported, such as discharge advice, telehealth, individual dietary counselling, participating in group education, commercial diet programs and online programs. |  |
| Cost effectiveness | **Does the cost-effectiveness of the intervention favour the intervention or the comparison?**  ○ Favours the comparison ○ Probably favours the comparison ○ Does not favour either the intervention or the comparison ○ Probably favours the intervention ○ Favours the intervention ○ Varies ● No included studies | The cost-effectiveness of high dietary fibre interventions for patients with diverticulitis as opposed to no intervention, or standard diets, has not been explored. However, cost-efficacy of high dietary fibre diet may be extrapolated from other studies, as a high dietary fibre diet is recommended for the general population and associated with a decrease in poor outcomes for a range of chronic conditions (NHMRC, 2005). Gordon et. al. (2007) found that overall dietary interventions, whether delivered by dietitians or other members of the multidisciplinary team, were cost-effective when considering improvements in a range of clinical outcomes related to diet in general. | Gordon, Louisa S. and Graves, Nicholas and Hawkes, Anna L. and Eakin, Elizabeth G. (2007) A Review of the Cost-Effectiveness of Face-to-Face Behavioural Interventions for Smoking, Physical Activity, Diet and Alcohol. Chronic Illness 3(2):pp. 101-129.  Nutrient Reference Values for Australia and New Zealand Including Recommended Dietary Intakes. 2005, National Health and Medical Resarch Council: Canberra. |
| Equity | **What would be the impact on health equity?**  ○ Reduced ○ Probably reduced ○ Probably no impact ● Probably increased ○ Increased ○ Varies ○ Don't know | Evidence suggests that people with low socio-economic status have a lower quality of diet, lower nutritional knowledge, and have less resources with which to engage support for dietary improvement (Darmon, et. al. 2008; Parmenter et. al. 2000). Therefore, programs should be targeted to support these disadvantaged populations in improving their diet quality to improve their overall health. | Darmon, N. and A. Drewnowski, *Does social class predict diet quality?* The American journal of clinical nutrition, 2008. **87**(5): p. 1107-1117.  Parmenter, K., J. Waller, and J. Wardle, *Demographic variation in nutrition knowledge in England.* Health education research, 2000. **15**(2): p. 163-174. |
| Acceptability | **Is the intervention acceptable to key stakeholders?**  ○ No ○ Probably no ● Probably yes ○ Yes ○ Varies ○ Don't know | Improvements in diet quality is a goal of all health care providers, health services and governments (Australian Dietary Guidelines, 2013; The EatWell Guide, 2016). In general, consumer groups also support this, but it cannot be assumed that all individual patients will be open to changing their diets (Marshall et. al. 2017). | Australian Dietary Guidelines. 2013, NHMRC, Australian Government: Canberra.  Choices, N., The Eatwell Guide. 2016.  Marshall, S., Reidlinger, D. P., Young, A., et al. 2017. The nutrition and food-related roles, experiences and support needs of female family carers of malnourished older rehabilitation |
| Feasibility | **Is the intervention feasible to implement?**  ○ No ○ Probably no ● Probably yes ○ Yes  ○ Varies ○ Don't know | There are existing systems by which long-term dietary support can be provided; and even though this is cost-effective in the long-term, the short-term costs may not be acceptable to the patient and/or the health services which may be under-resourced for dietetic staff. Therefore, feasibility varies significantly depending on who is providing the service. It should also be acknowledged that consuming a high dietary fibre diet does not present a significant expense to patients (<$1.50 to improve diet quality) (Rao et. al. 2013), and dietary supplements are not considered expensive. | Rao, M., Afshin, A., Singh, G., et al. 2013. Do healthier foods and diet patterns cost more than less healthy options? A systematic review and meta-analysis. BMJ open, 3**,** e004277. |

## Summary of judgements

|  | **Judgement** | | | | | | | **Implications** |
| --- | --- | --- | --- | --- | --- | --- | --- | --- |
| **Problem** | No | Probably no | **Probably yes** | Yes |  | Varies | Don't know | Probably favours high dietary fibre diet |
| **Desirable Effects** | Trivial | Small | Moderate | **Large** |  | Varies | Don't know | Probably favours high dietary fibre diet |
| **Undesirable Effects** | Large | Moderate | **Small** | Trivial |  | Varies | Don't know | Favours neither intervention |
| **Certainty of evidence** | **Very low** | Low | Moderate | High |  |  | No included studies | Probably favours high dietary fibre diet |
| **Values** | Important uncertainty or variability | Possibly important uncertainty or variability | Probably no important uncertainty or variability | **No important uncertainty or variability** |  |  |  | Probably favours high dietary fibre diet |
| **Balance of effects** | Favours the comparison | Probably favours the comparison | Does not favour either the intervention or the comparison | **Probably favours the intervention** | Favours the intervention | Varies | Don't know | Favours neither intervention |
| **Resources required** | Large costs | **Moderate costs** | Negligible costs and savings | Moderate savings | Large savings | Varies | Don't know | Probably favours high dietary fibre diet |
| **Certainty of evidence of required resources** | Very low | Low | Moderate | High |  |  | **No included studies** | Favours neither intervention |
| **Cost effectiveness** | Favours the comparison | Probably favours the comparison | Does not favour either the intervention or the comparison | Probably favours the intervention | Favours the intervention | Varies | **No included studies** | Probably favours high dietary fibre diet |
| **Equity** | Reduced | Probably reduced | Probably no impact | **Probably increased** | Increased | Varies | Don't know | Favours neither intervention |
| **Acceptability** | No | Probably no | **Probably yes** | Yes |  | Varies | Don't know | Probably favours high dietary fibre diet |
| **Feasibility** | No | Probably no | **Probably yes** | Yes |  | Varies | Don't know | Probably favours high dietary fibre diet |

**Recommendation:**

*Recommendation for the population:* Health care providers should recommend a long-term high dietary fibre intake (meeting or exceeding the nationally recommended intake for gender and age) after the acute episode of uncomplicated diverticulitis has resolved.

*Strength of the recommendation*: Strong recommendation for the intervention based on very low-quality body of evidence.

*Overall justification*: This review found low confidence in the evidence that high dietary fibre intake will result directly in improved risk for diverticulitis reoccurrence and/or gastrointestinal symptoms. However, a high dietary fibre diet is recommended as the standard diet for all adults by dietary guidelines [37, 38], and therefore this recommendation stands even though there is no strong confidence in added benefit for diverticulitis-related outcomes. This recommendation is considered strong, based on very low-quality-evidence, as potential benefits clearly outweigh risk, and support dietary guidelines [27]. It should be recognised that achieving sustained dietary change is difficult in western societies as it is not supported by the food environment [39], and therefore this diet should be recommended along with long-term support to achieve dietary change [40]. The evidence shows some improvement in clinical outcomes with the use of dietary fibre supplements with or without food-based dietary fibre increases; however, there is insufficient evidence to make specific supplementation recommendations [37, 41]. Some dietary guidelines do recommend nutrient supplementation in general if the nutrient target cannot be met through diet alone; therefore, this option should be considered on an individual basis [37]. Additionally, some patients have an intolerance to some forms of dietary fibre and/or other comorbidities which require a limitation or modification of dietary fibre intake [42]. These patients should receive individualised advice with follow-up to help achieve the best outcomes and management of potential ongoing symptoms.

*Subgroup considerations:* People with low-socioeconomic backgrounds are likely to require further support to achieve a high dietary fibre diet as this sub-group may have a poorer quality of diet at baseline, and have less resources to access dietary support services [43]. A high dietary fibre intake or specific high fibre foods may also be contraindicated in patients with additional comorbidities, particularly other diseases of the gastrointestinal tract or kidneys; and therefore, recommendations for these subgroups should be individualised.

*Implementation considerations*: Although the recommendation of achieving a high dietary fibre diet may easily be made, patients may not be aware of what constitutes a high fibre diet and how it can be achieved [44].

*Monitoring and evaluation:* Patients should be linked with dietary support services following discharge so they can be monitored and supported to achieve sustained dietary change [40].
